# Potential Valorization of Waste Tires as Activated Carbon-Based Adsorbent for Organic Contaminants Removal

**DOI:** 10.3390/ma15031099

**Published:** 2022-01-30

**Authors:** Kawthar Frikha, Lionel Limousy, Joan Pons Claret, Cyril Vaulot, Karin Florencio Pérez, Beatriz Corzo Garcia, Simona Bennici

**Affiliations:** 1 Institut de Sciences des Matériaux de Mulhouse, Université de Haute-Alsace, 15 Rue Jean Starcky, F-68057 Mulhouse, France; kawthar.frikha@uha.fr (K.F.); lionel.limousy@uha.fr (L.L.); cyril.vaulot@uha.fr (C.V.); 2 Sorigué, Ronda Guinardó, 99, 08041 Barcelona, Spain; J.Pons@sorigue.com (J.P.C.); k.florencio@sorigue.com (K.F.P.); b.corzo@sorigue.com (B.C.G.)

**Keywords:** waste tires, ground rubber tire, pyrolysis, char, activation, activated char, adsorption, wastewater treatment

## Abstract

The present study investigates the potential of waste tires to produce a valuable adsorbent material for application in wastewater treatment. In the first stage, the pyrolysis of ground rubber tire was explored using non-isothermal and isothermal thermogravimetric analysis experiments. The effect of operating parameters, such as heating rate and pyrolysis temperature, on the pyrolysis product yields was considered. The slow pyrolysis of ground rubber tire was taken up in a large-scale fixed-bed reactor for enhanced char recovery. Four pyrolysis temperatures were selected by thermogravimetric data. The product yields were strongly influenced by the pyrolysis temperature; at higher temperatures, the formation of more gases and liquid was favored, while at lower pyrolysis temperatures, more char (solid fraction) was formed. The produced chars were characterized in terms of mineral composition, textural properties, proximate analysis, and structural properties to identify the relationships between the pyrolysis temperature and the char properties. In a second step, a series of activated chars were prepared, starting from the pyrolytic chars via chemical and/or physical activation methods. Then, the activated chars were characterized and tested as adsorbents for atrazine and ibuprofen. Adsorption experiments in aqueous media were carried out in a small-scale batch reactor system. Chemical activation seems appropriate to significantly reduce the inorganic compounds initially present in ground rubber tire and contribute to an important increase in the surface area and porosity of the chars. Adsorption experiments indicated that chemically activated chars exhibit high aqueous adsorption capacity for atrazine.

## 1. Introduction

Over the past two decades, the increase in vehicle industry has generated a huge amount of waste tires [1]. About 4 billion tons of waste tires are generated every year, all over the world [2]. The European Union (EU), North America, and Southeast Asia take up 90% of the world’s waste tires generation [3]. The increase in the amounts of waste tires is well exemplified by the EU, in which more than 2.7 million tons of waste tires are generated every year [4]. To reduce the waste tires flow generation, the EU implemented council directive on end-of-life vehicles (2000), which provided measures and guidance on waste tires management [5]. The main routes for waste tires management are landfilling, energy recovery, material recovery, retreading, and reuse/export [6]. Meanwhile, the council directive on the landfill of waste (1999) banned the landfilling of waste tires by 2006 [7]. Tires are mainly composed of natural and/or synthetic rubber; a vulcanization agent such as sulfur; carbon black as a reinforcing filler; steel cords; textile fibers; and other additives, such as zinc oxide, as a vulcanization activator. The diverse composition of tires makes their energy and/or material recovery a profitable business. Moreover, the high calorific value (28–37 MJ kg^−1^) of tires, as well as the low mineral matter, make tires a potential non-fossil energy source [1]. However, some energy recovery routes, such as incineration or combustion, may lead to the emission of hazardous compounds and metals [4]. To minimize the negative impacts of waste management on the environment, such as those caused by the energy recovery routes, thermal recycling of waste tire materials through pyrolysis can be considered the most convenient practice [8]. Waste tires pyrolysis is an innovative alternative for waste tires management, producing fuel and other solid products in a more environmentally friendly manner. Indeed, lower amounts of hazardous gas, such as nitrogen oxides (NO_x_) and sulfur oxides (SO_x_), and better quality of the solid residue can be produced due to the inert atmosphere in the pyrolysis process [9]. During the pyrolysis process, the organic volatile matter of tires, mainly the rubber polymers, are decomposed to lower molecular weight products, oils, and gases, while the inorganic compounds and the nonvolatile carbon black, contained in the waste tire, remain as solid char residue [10]. Many research groups have demonstrated that pyrolytic oils can be used as fuels [11,12,13,14,15,16] or chemical feedstock [17,18,19], the gas can be used as a process fuel [20,21,22,23], and the char has the potential to be used as carbon black or activated carbon (AC) [24,25,26]. The yields and characteristics of pyrolysis products depend on the waste tires feedstock (composition and particle size) and the operating parameters, such as temperature, heating rate, vapors/solid residence time, and atmosphere. The pyrolysis of waste tires in an inert atmosphere at 300–900 °C has been studied by many researchers, and the effects of operating conditions on the product yields (gases, oils, and char) have been reported [12,27]. In a study [12], pyrolysis of waste tires was carried out in a fixed-bed reactor at 300–700 °C at a heating rate of 15 °C min^−1^. Char yield decreased when temperature increased from 300 to 500 °C, whereas liquid and gas yields increased. In another study [27], pyrolysis of waste tires was carried out in a fixed-bed reactor at 300–720 °C at a heating rate of 5 °C min^−1^. As the pyrolysis temperature increased from 300 to 720 °C, the char yields decreased from 94 to 39 wt.%, the oil yield increased from 3.6 to 55 wt.%, and the gas yield increased from 2.4 to 6.4 wt.%. Processing this carbonaceous residue into a high-value product, such as carbon-based adsorbent or catalyst support [28,29], is an interesting option. Chars derived from the pyrolysis of waste tires could be used as adsorbents for gas storage or pollution control applications. The surface area of an adsorbent is generally a key parameter when considering its adsorption properties. Chars derived from the pyrolysis of waste tires were found to have surface areas of 30–90 m^2^ g^−1^ [24,25,26,27], which are too low for use as an adsorbent as compared to a commercial carbon-based adsorbent. The activation process is often employed to improve the textural and chemical properties of carbon materials, using both heat and an activating agent. During activation, the activating agent diffuses into the char pores, causing a chemical reaction with the carbon atoms and loss of carbon. The reaction rate is influenced by multiple factors, such as the activating agent, the char composition, the ash content, the textural properties of the char, the reaction temperature, and the reaction time [30,31,32,33,34]. Basically, there are two different activation processes for the preparation of activated char: the so-called chemical and physical activation. Chemical activation consists of impregnation of the char with a chemical activating reagent, such as zinc chloride, phosphoric acid, sodium hydroxide, potassium carbonate, or potassium hydroxide, followed by carbonization of the impregnated char at temperatures between 600 and 850 °C, in an inert atmosphere [35,36,37,38]. Physical activation involves a thermal treatment of the char under an oxidizing atmosphere up to 1100 °C using gaseous oxidizing agents, such as carbon dioxide, steam, air, or a mixture of these [12,13,14].

The potential of waste tire-derived AC as adsorbents has been evaluated in both aqueous [39,40] and gas media [41,42], achieving adsorption capacities equivalent to those obtained with commercial AC or to those obtained at laboratory scale from other carbon sources, such as biomass waste [43,44], agri-food waste [45], textile waste [46], and municipal waste [47]. Concerning the environmental impact on the preparation of AC, to our knowledge, there are no published studies examining the environmental impact of the preparation of AC from ground rubber feedstock from a Life Cycle Assessment (LCA) perspective. However, some studies [48,49] on the environmental impact of the preparation of AC, using other types of waste as feedstock, are available in the literature. In one study [48], the authors have found that the environmental impacts of the preparation of AC, at laboratory scale, are mainly dominated by the activation process, specifically the impregnation step, due to the use of a chemical activation agent. The global warming potential impact was found to be 11 kg CO_2-_equivalent/kg AC. The steps involving feedstock production, feedstock logistics, and AC logistics presented low environmental impacts (0–6.4%). In another study [49], the authors demonstrated that the production of AC from brewery waste, at laboratory scale, complies with the ISO 14040 standard on LCA.

Regarding the industrial aspect of this study, the LCA on AC production, at laboratory and industrial scales, from waste tires is worth conducting to quantify the emission of hazardous materials, resources consumed, and the impact of the use of AC on the environment. From a qualitative point of view, the negative environmental impact of the preparation of AC from ground rubber feedstock can be managed, especially if certain modifications are incorporated into the production process of AC, such as recovery of KOH after AC washing, recovery of the energy from pyrolysis and activation hot gases, pyrolysis/activation gas recovery for reuse as an energy source, and regeneration of the used AC after water treatment application. Moreover, AC production using waste tires not only contributes to the development of materials for water environment treatment, but also contributes to the waste tires management. In addition, oil-based fuels production using waste tires provides increased benefits with respect to the development of alternative energy resources with low environmental impacts compared with nonrenewable fossil resources.

The aim of the present work is to study the potential valorization of an industrial waste, waste tires, as an adsorbent material for organic contaminants removal. For this purpose, the production of activated chars from waste tires was investigated via pyrolysis and activation process. The effects of the different treatments on the chemical and textural properties of the resulting carbon materials (char and activated chars) were investigated. The adsorption characteristics of the obtained activated chars in aqueous media were also investigated, where atrazine and ibuprofen were used as representative adsorbates.

## 2. Materials and Methods

### 2.1. Waste Tires Feedstock

Ground rubber with an average particle of 1.5–4 mm was used as the char precursor feedstock. The recycled ground rubber was supplied by Sorigué, Spain and produced from a combination of cars’ (60 wt.%) and trucks’ (40 wt.%) waste tires.

The used ground rubber is a standardized material classified following the ASTM D 5603 “Standard Classification for Rubber Compounding Materials—Recycled Vulcanized Rubber”. This classification covers the compounding material commercially known as recycled vulcanized particulate rubber.

**Figure 1:**
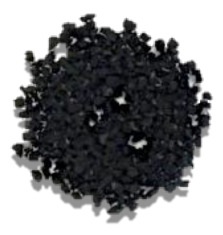
Picture of the ground rubber feedstock.

The picture of the ground rubber sample is shown in Figure 1. The characteristics of the ground rubber feedstock, including moisture content, volatile matter content, fixed carbon content, ash content, Element’s concentrations, and total polycyclic aromatic hydrocarbon concentration are presented in Table 1.

Proximate analysis was conducted according to the ASTM D7582-15 standard using a TOLEDO TGA/DSC 3+ thermogravimetric analyzer. About 15 mg of ground rubber sample were heated at a heating rate of 5 °C min^−1^ to 900 °C under a nitrogen atmosphere with a gas flow rate of 100 mL min^−1^ and held isothermally under a nitrogen atmosphere for 10 min. Then the atmosphere was switched to air (100 mL min^−1^) for 60 min. Element’s concentrations were determined by inductively coupled plasma mass spectrometry (ICP-MS) and inductively coupled plasma atomic emission spectrometry (ICP-AES), according to the NF EN ISO 11885, NF T 90-043, and NF EN ISO 17852 standards provided by LABOSPORT laboratory.

### 2.2. Thermogravimetric Analysis Experiments

Two sets of thermogravimetric analysis (TGA) experiments were carried out to evaluate the effect of heating rate and temperature on the char yield and thermal degradation behavior of the ground rubber sample. A TOLEDO TGA/DSC 3+ thermogravimetric analyzer was used for all the experiments. Experimental protocol details and operating conditions are given in Table 2. For the non-isothermal experiments, the ground rubber samples were heated from 35 to 900 °C at a chosen heating rate (5, 10, and 20 °C min^−1^), followed by a 10 min hold at 900 °C and then a 60 min hold at 900 °C in an oxidizing environment. For the isothermal experiments, first, the samples were heated from 35 °C to the chosen temperature (450, 500, 550, and 600 °C) with a heating rate of 5 °C min^−1^ and then kept under isothermal conditions for 180 min. Second, the samples were heated from the isothermal temperature to 900 °C, followed by a 60 min hold at 900 °C and then a 10 min hold at 900 °C in an oxidizing environment. For all the experiments, the gas flow rate was 100 mL min^−1^, the ground rubber sample was used as received, and the initial mass sample was about 10−15 mg. For each experiment, the proximate analysis data—volatile matter, fixed carbon, and ash content—were determined.

### 2.3. Pilot-Scale Pyrolysis Experiments

Pilot-scale pyrolysis experiments were carried out in a fixed-bed reactor, externally heated by an electrical furnace. The reactor was fully instrumented in terms of gas flow control, furnace temperature control, and temperature monitoring throughout. A condenser system was used to trap the condensable gases (liquids). Non-condensable gases were sent into a carbon filtration unit before reaching the atmosphere. Three pyrolysis assays were performed under a nitrogen atmosphere with the same solid residence time at the temperatures of 450, 500, and 550 °C. Since the water content of the ground rubber was too low, there was no need to dry the sample before the pyrolysis experiments. Before the pyrolysis experiment, the reactor was purged with a nitrogen gas flow of 10 L min^−1^ for 10 min to remove air. In a typical pyrolysis experiment, approximately 2.6 kg of ground rubber sample were introduced into the reactor, heated at a controlled rate of 5 °C min^−1^ to the desired pyrolysis temperature, and held at that temperature for 3 h. During the pyrolysis experiment, the reactor was purged continuously with nitrogen at a fixed flow rate of 10 L min^−1^ to remove away the evolved gases from the reaction zone, thus reducing the eventual secondary reactions, such as thermal cracking, repolymerization, and recondensation. After completion of the pyrolysis, the reactor was left to cool down, and the solid residue was recovered from the reactor and weighed to determine the mass of the char produced. The char samples obtained at the three pyrolysis temperatures were labeled as BC450, BC500, and BC550. The condensed liquid collected at the end of the experiment was weighed to determine the mass of the oil (tar) product. The char, oil, and gas product yields were calculated relative to the initial sample mass using Equations (1)–(3):

(1) $$\mathrm{Char}\;\mathrm{yield}\;\left(\mathrm{wt}.\;\%\right)=\frac{\mathrm{Mass}\;\mathrm{of}\;\mathrm{char}\;}{\mathrm{Mass}\;\mathrm{of}\;\mathrm{sample}}\times 100$$

(2) $$\mathrm{Tar}\;\mathrm{yield}\;\left(\mathrm{wt}.\%\right)=\frac{\mathrm{Mass}\;\mathrm{of}\;\mathrm{tar}}{\mathrm{Mass}\;\mathrm{of}\;\mathrm{sample}}\times 100$$

(3) $$\mathrm{Gas}\;\mathrm{yield}\;\left(\mathrm{wt}.\;\%\right)=100-\mathrm{Char}\;\mathrm{yield}-\mathrm{Tar}\;\mathrm{yield}$$

### 2.4. Activation Experiments

**Figure 2:**
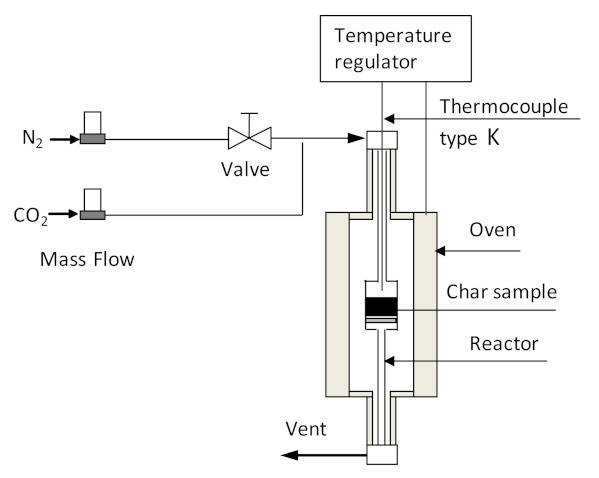
Scheme of installation used for activation experiments.

The char obtained at a pyrolysis temperature of 450 °C (BC450) was selected as precursor for activated char production. Prior to activation, the char sample was sieved to 1.5–4 mm particle size. In this work, we adopted three activation methods to generate the activated char, including chemical activation (CA), physical activation (PA) and combined chemical–physical activation (CPA) (chemical activation followed by physical activation). The chemical activation experiment involved the impregnation of the char with potassium hydroxide (KOH, ≥85%, Fluka, Steinheim, Germany) as the activating agent, followed by heating the chemically impregnated char at 700 °C for 3 h in a nitrogen-purged quartz-tube reactor, externally heated by an electric vertical furnace. The scheme of the reactor used for activation experiments is shown in Figure 2. After heat treatment, the activated char was cooled under nitrogen purge and then washed and filtered several times with distilled water until the pH of the filtrate was around 7. Finally, the washed sample was dried at 105 °C for 24 h in air. Physical activation was carried out in a quartz-tube reactor using carbon dioxide as the activating agent. A char sample of about 3 g was introduced into the reactor (Figure 2) and heated from an ambient temperature to 850 °C under nitrogen flow (100 mL min^−1^) at a heating rate of 5 °C min^−1^. When the target activation temperature was reached, the inert atmosphere was substituted by carbon dioxide with a flow rate of 100 mL min^−1^. The reactor temperature was held at 850 °C for 2 h and then cooled to room temperature under a nitrogen atmosphere. For the combined chemical–physical activation experiment, the activated char produced by chemical activation experiment was subjected to subsequent physical activation, in which time activation lasted only for 20 min. The activated char was weighed to calculate the activated char production yield. The activated char yield was calculated using equation 4. The activated char samples obtained through chemical, physical, and combined chemical–physical activations were labeled as CA-BC450, PA-BC450, and CPA-BC450, respectively.

(4) $$\mathrm{Activated}\;\mathrm{char}\;\mathrm{yield}\;\left(\mathrm{wt}.\;\%\right)=\frac{\mathrm{Mass}\;\mathrm{of}\;\mathrm{activated}\;\mathrm{char}}{\mathrm{Mass}\;\mathrm{of}\;\mathrm{char}}\times 100$$

### 2.5. Chemical and Physical Characterization

Physical and chemical characterization were performed on the char and activated char samples to gain insight into the properties of tire-derived char sorbents. Such properties include proximate analysis, ultimate analysis, surface area and micropore volume analysis, point of zero charge (PZC) measurements, scanning electron microscopy (SEM), energy dispersive X-ray spectrometry (EDX), X-ray diffraction analysis (XRD) and Raman spectroscopy.

Proximate analysis was conducted according to the ASTM D7582-15 standard using a TOLEDO TGA/DSC 3+ thermogravimetric analyzer. An approximately 20 mg sample were heated at 5 °C min^−1^ to 900 °C under a nitrogen atmosphere with a constant gas flow rate of 100 mL min^−1^ and held isothermally under the nitrogen atmosphere for 10 min. Afterwards, the gas flow was switched from nitrogen to air, and the temperature was held at 900 °C for 60 min. Weight loss up to 105 °C represents moisture content. The weight loss between 105 and 900 °C represents the volatile matter content. The residue remaining after combustion in the air at 900 °C represents ash. Fixed carbon content was determined by difference.

Surface area and micropore volume analysis was conducted on a Micromeritics ASAP2420 volumetric gas adsorption apparatus using N_2_ or CO_2_ as a gas adsorbate at −196 °C and 0 °C, respectively. Before gas adsorption measurements, the samples were outgassed under vacuum at 200 °C for 10 h. Surface area and micropore volume were determined by applying Brunauer-Emmet-Teller (BET) and t-plot methods, respectively. Total pore volume and average pore diameter were determined using the standard Barrett-Joyner-Halenda (BJH) method applied to the desorption branch of the nitrogen isotherm.

Ultimate analysis was performed with a Wavelength Dispersive X-ray Fluorescence (WDXRF) using a PANalytical Zetium (4 kW) spectrometer. Before analysis, pellets of the samples (13 mm diameter and 1 mm thick) were obtained by pressing the powders (100 mg) under 9-ton pressure during 10 min.

Characterization of the carbon matrix’s microstructure was conducted using scanning electron microscopy, Raman spectroscopy, and X-ray diffraction analysis. SEM micrographs were acquired on a JSM 7900 JOEL microscope at 5 kV accelerating voltage. XRD analyses were carried out using a PANalytical MPD X’Pert Pro diffractometer operating with Cu K α radiation, λ = 0.15406 nm at 40 mA and 45 kV. Data were recorded at room temperature, applying 2 theta (2θ) scanning range of 10–70° and a step size of 0.017° with a scan step time of 220 s. Raman spectra were recorded with a BX40 LabRam spectrometer equipped with a CCD camera detector. The spectra were acquired with a 50× lens, an excitation laser operating at a wavelength of 532 nm, and an acquisition time of 180 s.

The point of zero charge (PZC) of the activated chars, known as pH_PZC_, corresponds to the pH value at which the surface has a zero net charge and was determined by the pH drift method. At pH < pH_PZC_, the char surface has a net positive charge, while at pH > pH_PZC,_ the surface has a net negative charge. This method consisted of preparing six solutions with initial pH values between 3 and 13 by diluting aqueous solutions of 0.1M NaOH or 0.1 M HCl. Solutions of 50 mL of each were prepared and piped into glass screw-capped bottles, and 0.1 g of the activated char was added to each solution. Then, the suspensions were agitated for 48 h and filtered. The final pH of each filtrate was measured using a digital pH meter and plotted against the initial pH. The pH_PZC_ point was determined at the point in which pH_final_ is equal to pH_initial_.

Before characterization, char and activated char samples were dried at 105 °C for 24 h and saved in a desiccator to prevent moisture absorption.

### 2.6. Aqueous Phase Adsorption Tests

BC450, CA-BC450, PA-BC450, and CPA-BC450 samples were selected as adsorbents to perform adsorption tests. Atrazine (C_8_H_14_ClN_5_, ≥98%, Sigma-Aldrich, Steinheim, Germany) and ibuprofen (C_13_H_18_O_2_, ≥98%, Sigma-Aldrich) were chosen as adsorbates. These organic micro-pollutant adsorbates were used as received without any further purification, and their molecular sizes and structures, which were estimated using ACD/ChemSketch software, are shown in Table 3.

Prior to adsorption tests, the activated carbons were dried overnight in a ventilated oven at 110 °C. Equilibrium adsorption experiments were performed in ambient conditions in separate batch systems, each containing different concentrations of atrazine or ibuprofen. First, stock solutions of 25 mg L^−1^ atrazine or 20 mg L^−1^ ibuprofen were prepared by dissolving an appropriate amount of atrazine or ibuprofen in deionized water. Then, four solutions of various initial concentrations were prepared from these stock solutions by sequential dilution using deionized water. The concentration range of each adsorbate was from 5 to 20 mg L^−1^ (5, 10, 15, and 20 mg L^−1^).

Equilibrium adsorption experiments for each adsorbate were performed by transferring an adequate pre-weighed amount of the dry adsorbent into a set of 50 mL screw-capped polypropylene centrifuge tubes used as batch reactor systems, each containing 40 mL of adsorbate solution of different concentrations. The sealed tubes were agitated mechanically at a uniform shaking speed for 48 h in a horizontal mechanical shaker. This shaking period was sufficient for establishing adsorption equilibrium. The adsorption tests were determined without adding any electrolyte solution into the system to avoid any interference of additional substances in the system. After shaking, the suspensions were filtered through 0.45 μm syringe filters, and the residual atrazine or ibuprofen concentration in the filtrate was determined using a Perkin Elmer Lambda 35 UV–Visible spectrophotometer. The absorption spectra were recorded from 200 to 900 nm with 1 nm bandwidth, and the absorbance for each solution was measured at 222 nm (wavelength of maximum absorption). Calibration curves of atrazine and ibuprofen, at different concentrations, were generated by fitting the absorbance as a function of the concentration at the maximum wavelengths of 222 nm. The calibration equations of atrazine and ibuprofen were calculated by linear regression of the calibration curves through the representation of the adsorbate concentration versus the absorbance. The resulting calibration lines presented good determination coefficients (R^2^) of at least 0.998.

The adsorption capacity at equilibrium (q_e_, mg g^−1^), corresponding to the amount of atrazine or ibuprofen adsorbed per gram of activated char, and the adsorption efficiency (%), corresponding to the percentage removal of pollutants by the char or activated char, were calculated using Equations (5) and (6).

(5) $$\mathrm{Adsorption}\;\mathrm{capacity}\;\left(q_{e},{\;\mathrm{mg}\;g}^{-1}\right)=\frac{(C_{0}-C_{e})\times V}{m}$$

(6) $$\mathrm{Adsorption}\;\mathrm{efficiency}\;\left(\%\right)=\frac{(C_{0}-C_{e})\times 100}{C_{0}}$$

where C_0_ (mg L^−1^) is the initial concentration of solute, C_e_ (mg L^−1^) is the concentration of solute at equilibrium, V (L) is the volume of the aqueous solution, and m (g) is the mass of the char/activated char.

The adsorption data of atrazine and ibuprofen were fitted to both Langmuir and Freundlich isotherm models [50,51,52,53,54]. The Langmuir isotherm is commonly applied to monolayer adsorption on a homogeneous adsorbent surface (monolayer adsorption model), and it is given by Equation (7). The constants q_max_ and K_L_ can be determined from its linearized form (plot of C_e_/q_e_ vs. C_e_):

(7) $$q_{e}=\frac{q_{\max}K_{L}C_{e}}{\left(1+K_{L}C_{e}\right)}$$

where q_max_ (mg g^−1^) is the maximum adsorption capacity, and K_L_ (L mg^−1^) is the Langmuir constant.

The Langmuir equation can be linearized to calculate the parameters q_max_ and K_L_ using Equation (8) [55] (plot of C_e_/q_e_ vs. C_e_):

(8) $$\frac{C_{e}}{q_{e}}=\frac{1}{q_{\max}K_{L}}+\frac{C_{e}}{q_{\max}}$$

The Freundlich isotherm is employed for adsorption surfaces with nonuniform energy distribution (multilayer adsorption model), and it is given by Equation (9):

(9) $$q_{e}=K_{F}C_{e}^{\frac{1}{n}}$$

where K_F_ (mg g^−1^) is the Freundlich adsorption capacity, and 1/n (dimensionless) is the Freundlich constant related to the surface heterogeneity.

The Freundlich equation can be linearized to calculate the parameters K_F_ and n using Equation (10) (plot of ln(q_e_) vs. ln(C_e_)). The adsorption can be considered favorable when 0 < 1/n < 1 [51]:

(10) $$\ln\left(q_{e}\right)=\frac{1}{n}\ln\left(C_{e}\right)+\ln(K_{F})$$

## 3. Results and Discussion

### 3.1. Thermogravimetric Analysis

**Figure 3:**
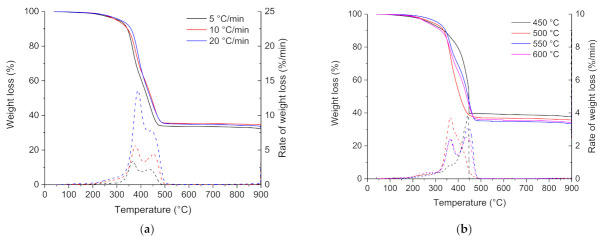
TG and DTG curves for the pyrolysis of the ground rubber sample at different (**a**) heating rates and (**b**) pyrolysis temperatures.

Influence of heating rate: Thermogravimetric (TG) and differential thermogravimetric (DTG) curves for the pyrolysis of the ground rubber sample at heating rates of 5, 10, and 20 °C min^−1^ are shown in Figure 3a. The ground rubber sample decomposed similarly at the three different heating rates; the thermal degradation started at around 200 °C, and the mass decreased steadily between 300 and 500 °C. Pyrolysis of the ground rubber sample at the different heating rates was mainly complete by 500 °C, above which there is no further weight loss. The pyrolysis residue increased slightly with the increasing heating rate, and it represented an average of 33 wt.% of the initial weight. The lowest heating rate (5 °C min^−1^) provided higher residence time and resulted in a more extended degradation than at higher heating rates. According to DTG curves, the ground rubber sample loses weight in a broad temperature range between 200 and 550 °C and yields three DTG peaks. This is consistent with the relatively complex chemical composition of tire rubber. According to the previous studies [56,57,58,59,60,61], the lower temperature between 200 and 300 °C represents the decomposition of oils, plasticizer, and additives embodied in the tire rubber and the second (300–400 °C) and third (400–500 °C) regions of weight loss represent the decomposition of natural rubber (NB), polybutadene rubber (BR), and polybutadene-styrene rubber (SBR).

Proximate analysis data obtained from the pyrolysis of the ground rubber sample at different heating rates are shown in Table 4. Results showed that the distribution of the different volatile matter, fixed carbon, and ash fractions was roughly unaffected by heating rate. The lowest heating rate (5 °C min^−1^) generated the highest volatile matter fraction and the lowest fixed carbon fraction. Pyrolysis at a low heating rate may provide a higher residence time of the sample at the reaction temperature range, which results in greater devolatilization and more release of volatiles.

Influence of pyrolysis temperature: TG and DTG curves for the pyrolysis of the ground rubber sample at temperatures of 450, 500, 550, and 600 °C are shown in Figure 3b. For the four pyrolysis runs, the decomposition occurred in the same temperature range between 200 and 500 °C. The char yield decreased slightly with increasing pyrolysis temperature. Char yields from pyrolysis of the ground rubber sample at temperatures between 450 and 600 °C were in the 35–40 wt.% range. This is consistent with the results of other workers [12,27,62,63]. The temperatures for which the maximum rate of reaction occurred (T_1_ and T_2_) are almost identical for the four runs. The char yield decreased as the pyrolysis temperature increased and reached a minimum yield of 34.8 wt.% at 550 °C and then increased as the temperature increased from 550 to 600 °C. The minimum yield of char and maximum yield of volatiles at 550 °C can be attributed to the balance for the competition between the primary pyrolysis reaction and secondary post-cracking reaction.

Proximate analysis (Table 4) for the pyrolysis of the ground rubber sample at different temperatures gave approximately a fixed carbon content between 26 and 36 wt.%, a volatile matter content between 60 and 65 wt.%, and an ash content between 1 and 13 wt.%. The volatile matter and fixed carbon contents increased with the increase in temperature from 450 to 550 °C. The further increase in pyrolysis temperature from 550 to 600 °C led to a decrease in both volatile matter and fixed carbon yields. Conversely, the increase in pyrolysis temperature was found to have the opposite effect on the evolution of ash content; the ash content underwent a remarkable decrease during the increase in temperature from 450 to 550 °C, and it increased considerably at 600 °C. The significant change in ash content can be explained by the evolution of the chemical composition of inorganic matter, initially present in the tire, upon increasing the pyrolysis temperature. Tire-rubber is mainly composed of natural and/or synthetic rubber, sulfur, and other mineral additives, such as zinc oxide. During pyrolysis, the inorganic species initially present in tires are prone to volatilization, decomposition, and recombination, or they stay stable as the organic component of tire decomposes. The occurrence of these reactions is mainly determined by the pyrolysis temperature and the chemical composition of the sample taken for analysis (sampling).

The present trend of ash content evolution was comparable with another tested ground rubber sample with an average particle size of 0.2–0.63 mm. However, this sample showed lower variations in ash content upon the increase in the pyrolysis temperature. These results indicated that the ground rubber sample with large particle size exhibited constitutional heterogeneity, which means that the particles do not have a strictly identical chemical composition.

### 3.2. Product Yields

Effect of pyrolysis temperature on product yields: The product distributions from pyrolysis of the ground rubber sample at different temperatures are presented in Table 5. The pyrolysis product distribution did not change significantly as the temperature increased from 450 to 550 °C. The volatiles (liquids and gases) yields are almost constant at a value of about 62 wt.%. The solid yield remained essentially constant with a mean of 38 wt.%. The data suggested that the primary pyrolysis reaction was already completed in the pilot-scale fixed-bed reactor at a temperature of 450 °C. The yield of pyrolytic oil increased from 29.7 to 34.2 wt.%, whereas the gas yield decreased with increasing temperature from 450 to 550 °C. Similar results have been obtained by other researchers [12,64,65,66]. Rodriguez et al. [12] carried out pyrolysis experiments on 2–3 cm wide cross-sections of car tires at 300, 400, 500, 600, and 700 °C at a heating rate of 15 °C min^−1^. The authors have found that pyrolysis was incomplete at 300 and 400 °C, and the solid yield dropped by 32 wt.% over the 300–500 °C temperature range. However, no significant influence of temperature on solid yields was observed over 500 °C, and the solid yields obtained at 500, 600, and 700 °C were around 44 wt.%. In another study, Li et al. [64] carried out pilot-scale pyrolysis experiments on 13–15 mm shredded scrap tires using a continuous pyrolytic rotary kiln reactor and varying the pyrolysis temperature from 450 to 650 °C. The authors have found that the solid char yield remained essentially constant with a mean of 39.8 wt.%, except for the relatively high value of 43.9 wt.% at a temperature of 450 °C, which was higher than the data in this study. The char yield in the pilot-scale static bed reactor was compared with that of other reactor processes in Table 5. The products recovered by the different pyrolysis processes in the 400–600 °C temperature range were 30.0–55.9 wt.% pyrolytic char, 24.8–65.0 wt.% oil, and 30.0–55.9 wt.% gas fraction. The data suggest that the pyrolytic char yield is not sensitive to the heating rate, behavior that differs greatly from the pyrolysis of biomass, for which the biochar yield strongly depends on the heating rate [67]. Conversely, the oil yield varied greatly for the different reaction conditions.

Effect of activation method on activated char yields: The yields of activated chars are presented in Table 6. Data reported in the literature are also given for comparison. Activation of the char at 700 °C with KOH yields 70 wt.% of activated char. Activation of the char at 850 °C with CO_2_ yields 87 wt.% of activated char. A large drop in the yield from 70 to 57 wt.% was observed when the chemically activated char was subjected to a subsequent physical activation. Work reported elsewhere had shown that the activation treatment, under very close conditions, yields 63–93 wt.% of activated char. Indeed, Acosta et al. [68] obtained chemically activated char yields of 81 and 74 wt.% at activation temperatures of 700 and 750 °C, respectively. Ucar et al. [69] reported an activated char yield of 93 wt.% at a temperature of 900 °C while Helleur et al. [40] and Danmaliki et al. [70] obtained activated char yields of 88 and 62 wt.% at activation temperatures of 875 and 850 °C, respectively.

### 3.3. Characterization of the Chars

**Figure 4:**
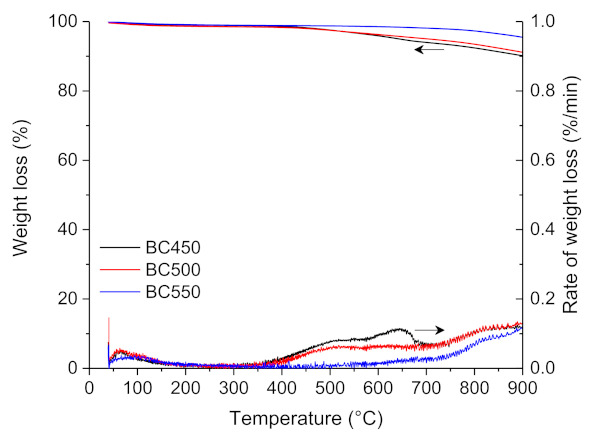
TG and DTG curves for the pyrolysis of the ground rubber-based chars.

The thermogravimetric analysis of BC450, BC500, and BC550 char samples obtained through the pilot-scale pyrolysis experiments are shown in Figure 4. The TG curves of the three char samples showed a slight weight loss of 1–2 wt.% in the temperature range of 35–120 °C, which is due to moisture desorption. A very slight weight loss was observed in the BC550 thermogram with an increase in temperature to 900 °C, while a higher weight loss was observed for BC450 and BC500 samples between 400 and 900 °C, which can be ascribed to the incomplete pyrolysis of the ground rubber.

Table 7 illustrates the proximate analysis of the ground rubber-based chars. Fixed carbon content ranged from 78 to 86.5 wt.%, the volatile matter content ranged from 4.1 to 9.3 wt.%, and the ash content ranged from 9.4 to 12.7 wt.%. The volatile matter decreased with increasing pyrolysis temperature, and this can be attributed to the promoted devolatilization process with an increase in pyrolysis temperature from 450 to 550 °C. The high ash content of ground rubber-based biochar originated from the presence of high amounts of inorganic materials, such as zinc and copper, and their concentration in the final char via the pyrolysis process.

Table 7 lists the elemental analysis of the ground rubber-based chars at the different pyrolysis temperatures. The carbon content of the char samples ranged from 87 to 91 wt.%, which makes these materials good candidates for activated carbon production. The carbon content appears to be high when compared with biomass-based biochar and agricultural waste-based biochar [71,72]. The high carbon content is attributed to the application of carbon black as a reinforcing filler in rubber elastomers manufacturing [73]. Silicon, sulfur, and zinc constituted the major inorganic elements found in the char samples. These results are expected since Si, S, and Zn oxide are widely used as a reinforcing filler in rubber elastomers manufacturing, in addition to carbon black. The presence of sulfur can promote the adsorption of certain pollutants, such as heavy metals, onto the carbonaceous materials. Many studies have shown positive effects of sulfur groups on the adsorption properties of activated carbons [74,75,76]. No significant change was detected in the mineral composition, except silicon, between the chars produced at different pyrolysis temperatures. Silicon content showed a significant decrease from 2.81 to 0.63 wt.% when the pyrolysis temperature increased from 450 to 550 °C.

**Figure 5:**
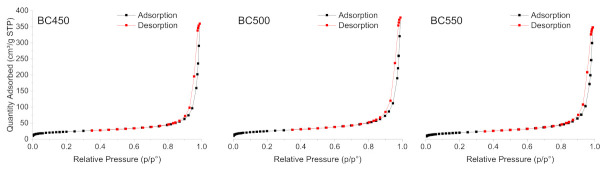
N_2_ adsorption-desorption isotherms of the ground rubber-based chars.

The N_2_ adsorption-desorption isotherms of the ground rubber-based chars produced at different pyrolysis temperatures are shown in Figure 5. All the char samples gave type (IV) isotherms, characteristic of mesoporous solids according to the IUPAC classification [77], with H1-type hysteresis loops. The BET surface area, total pore volume, and average pore width of the char samples are listed in Table 7. The BET surface area of the char samples varied from 71.41 (for BC550) to 85.88 m^2^ g^−1^ (for BC500). The pore volume ranged between 0.53 and 0.58 cm^3^ g^−1^ and followed a similar variation trend to the surface areas. All the pores were in the meso-range, and the average pore width varied from 34.05 to 36.61 nm. The BC500 sample presented the highest surface area, as well as the highest pore volume. The textural properties of the produced chars from ground rubber are similar to those recorded by other authors [25], and they do not show any great variations with the increase in pyrolysis temperature. Compared to other carbonaceous materials used as adsorbents, rubber-based chars with low surface areas cannot be used as an efficient adsorbent for the removal of pollutants. Therefore, an activation treatment should be applied to develop the porosity in the structure of the produced chars.

**Figure 6:**
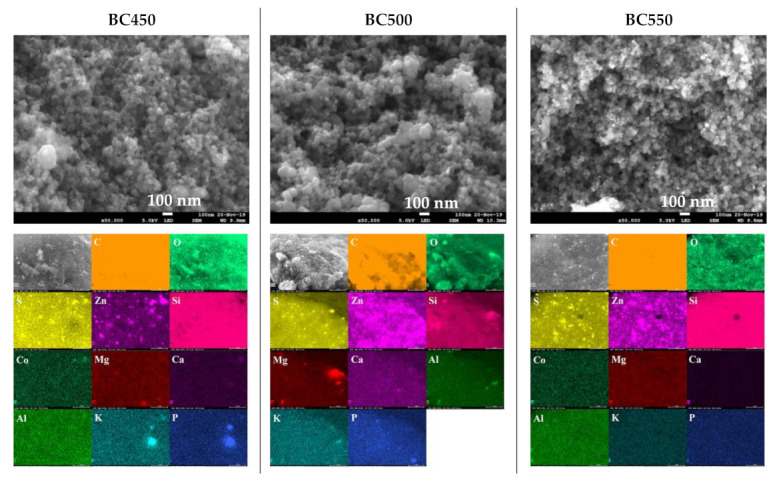
SEM images and elemental distribution maps of the ground rubber-based chars.

The SEM images, at a magnification of 50,000× and the EDX elemental maps of the ground rubber-based chars are given in Figure 6. SEM images revealed dense and rough surfaces without notable cracks or pores. Amorphous and quasi-spherical clusters of nano-sized particles can be seen, which could be attributed to the organic matter. The surface morphology in overall samples seemed not to be affected by increases in the pyrolysis temperature. EDX analyses of the char samples indicated the presence of various mineral elements, mainly S, Zn, Si, Co, Mg, Ca, Al, K, and P. The above results are in good agreement with the results obtained from XRF. For the BC450 and BC550 samples, lumps of zinc and sulfur grains existed at the same locations, which suggests that they occur in the form of zinc sulfide. Zinc sulfide might be produced by the reaction between the zinc oxide and sulfur contained in tires [63].

**Figure 7:**
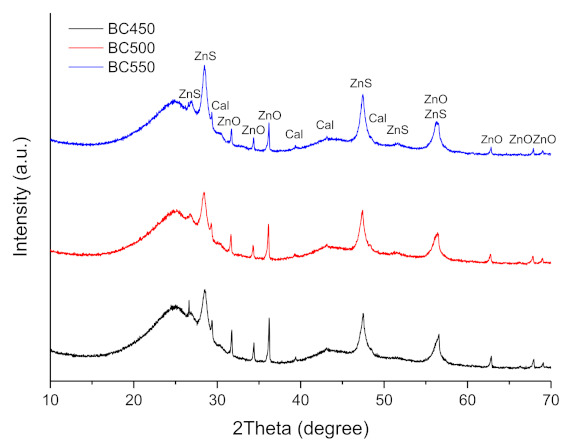
XRD patterns of the ground rubber-based chars.

The XRD patterns of the ground rubber-based chars are shown in Figure 7. The XRD patterns showed the presence of rhombohedral CaCO_3_ (JCPDS 01-078-4614), hexagonal ZnO (JCPDS 01-078-2585), and rhombohedral ZnS (JCPDS 01-089-2203). ZnO and sulfur present in the tires may react during pyrolysis to form ZnS. The diffraction peaks corresponding to the CaCO_3_ phase lose their intensity during an increase in the pyrolysis temperature from 450 to 550 °C, which is probably due to the decomposition reaction of calcium carbonate. The intensity of the baseline of the diffractogram at 2θ ≈ 25° is mainly related to the presence of non-aromatic amorphous carbon in the char samples [78].

The Raman spectra (data are not shown) of the different chars exhibited two relatively broad Raman bands at around 1350–1385 cm^−1^ and 1585–1590 cm^−1^. These bands correspond to the D-band and G-band, respectively. The G-band and D-band correspond to graphite and defects in the graphite carbon structure, respectively [79]. Structural parameters, such as the intensity ratios between the D- and G-bands (ID/IG ratio) for the three char samples were determined. The ID/IG ratio (see Table 7) of the ground rubber-based chars showed an increase with increasing pyrolysis temperature from 450 to 550 °C. The observed results suggest that the variation of ID/IG ratio in the studied temperature range is mainly related to the development of amorphous carbon, and apparently not to graphitization. Increasing the pyrolysis temperature above 550 °C might result in a decrease in ID/IG ratio due to the increase in the degree of graphitization (char evolution structure from the amorphous carbon to an organized carbon).

### 3.4. Characterization of the Activated Chars

Table 8 lists the concentrations of carbon, oxygen, and mineral elements in the activated chars. The mineral compositions of the chemically activated char were different from those determined for the non-activated chars. Al, Si, S, and Zn contents were lower in the chemically activated chars, and K and Mg slightly increased. This suggests that chemical activation with KOH could be considered an indirect demineralization treatment that decreases the mineral impurities in char, which will improve the properties and market value of the activated chars. The very low mineral content of the rest of the analyzed elements is the reason for not seeing any significant effects from the activation treatments. The mineral contents in the CO_2_-activated char were in the same range as found in non-activated char.

**Figure 8:**
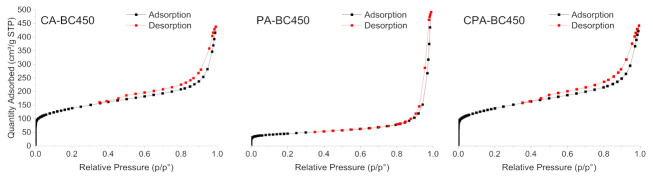
N_2_ adsorption–desorption isotherms of the activated chars.

Figure 8 shows the N_2_ adsorption–desorption isotherms of the activated chars obtained through the different activation methods. The development of micropores and mesopores for the chemically activated chars can be clearly deduced by the shape of the isotherms. CA-BC450 and CPA-BC450 samples exhibited isotherms of mixed types IV and I, with H4-type hysteresis loops that can be associated with the presence of narrow, slit-like pores. These isotherms are characteristic of adsorbents containing both micropores and mesopores, according to the IUPAC classification [77]. The N_2_ adsorption–desorption isotherm of the PA-BC450 sample is of mixed types II and I, typical for macroporous and microporous adsorbents, with an H3-type hysteresis loop related to aggregates of plate-like particles, giving rise to slit-shaped pores. The textural properties of the activated chars are summarized in Table 8. The activated chars exhibited a larger surface area than that of the non-activated char. The BET surface areas of the KOH-activated char according to N_2_ and CO_2_ adsorption measurements were 494 (six times higher than that of the non-activated char) and 309 m^2^ g^−1^, respectively. The CO_2_-activated char possesses a BET surface area of 164 m^2^ g^−1^ according to N_2_ adsorption measurements (two times higher than that of the non-activated char) and 117 m^2^ g^−1^ according to CO_2_ adsorption measurements. The BET surface areas, determined by N_2_ and CO_2_ adsorption measurements, of the char activated by both KOH and CO_2_ were in the same range as the surface areas of the char activated only by KOH. The results clearly demonstrated that the activation process is efficient for increasing the surface area and the development of new pores with different widths and volumes. A comparison of BET surface areas of activated chars produced in the present study with those reported in the literature is presented in Table 9. The surface areas of the produced activated chars are comparable with those reported in the literature. Activated chars with surface areas in the range of 166–405 m^2^ g^−1^ have been obtained from ground rubber by chemical activation using KOH as an activating agent. CO_2_-activated char with surface areas in the range of 91–399 m^2^ g^−1^ were also obtained.

**Figure 9:**
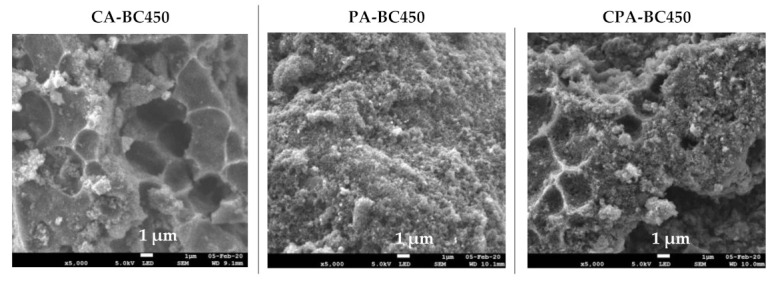

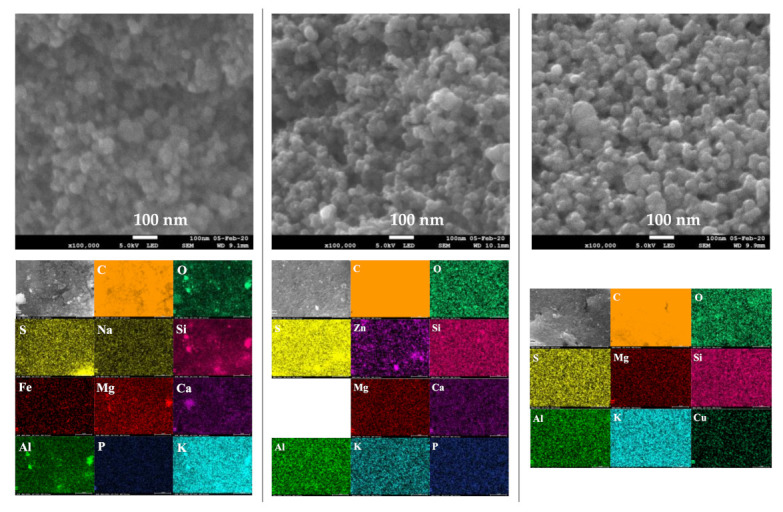
SEM images and elemental distribution maps of the activated chars.

The SEM images at two magnifications (5000× and 100,000×) and elemental distribution maps of the activated chars are given in Figure 9. The SEM images of the CO_2_-activated chars revealed dense and planar surfaces without notable cracks or pores. The SEM micrographs of the char activated by KOH and by both KOH and CO_2_ showed pits and hollows, as well as sharply defined cliffs denoted by curved lines. It is evident that chemical activation led to a significant surface topographical change of the char, which may result in increased adsorption capacity. Examination of SEM micrographs at high magnification (100,000×) shows that activated chars have the same morphology as those of non-activated char. Indeed, quasi-spherical clusters of nano-sized particles have been observed in all samples. Elemental mapping images showed uniform and homogenous distribution of mineral elements on the surfaces of the activated chars.

The D and G bands were in the expected regions of 1350–1385 cm^−1^ and 1585–1590 cm^−1^, respectively (data are not shown). The ID/IG ratios, determined from the Raman spectra, are listed in Table 8. The activated chars exhibited comparable band intensity ratios. An increase in band intensity ratio was observed after the activation process.

**Figure 10:**
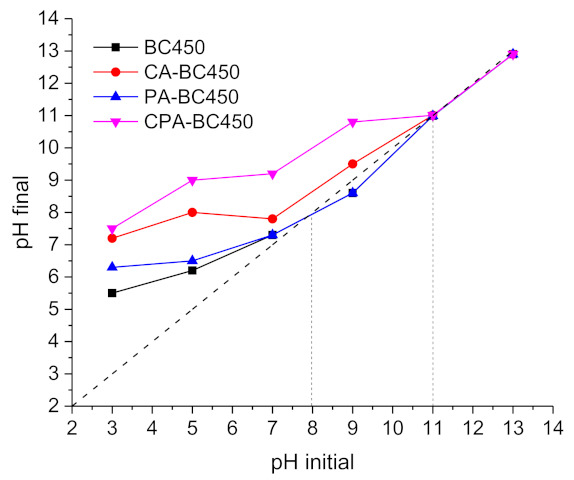
The pH of the point of zero charge (pH_PZC_) of the activated chars.

The final pH values (pH_f_) of the filtrates were plotted against the initial pH values of the prepared solution (pH_i_) to determine the surface character of the activated chars, acidic or basic. The results (Figure 10) show that most of the pH_f_ = f(pH_i_) curves of the activated chars are situated above the middle diagonal line (pH_i_ = pH_f_), indicating the basic character of their surface (negative surface charge). In general, the acidic character of carbon surfaces is linked to the presence of oxygen surface complexes or oxygen functionalities, such as carboxyl, lactones, and phenol, while the basic character is associated with the presence of oxygen atoms forming basic functional groups, such as pyrene, chromene, quinone or with the presence of p electrons in the graphene structure [72]. The pH_PZC_ (the pH at which the surface charge of the material is zero) of the BC450 and PA-BC450 samples was 8, while the pH_PZC_ of the CA-BC450 and CPA-BC450 samples was 11.

### 3.5. Adsorption Tests

**Figure 11:**
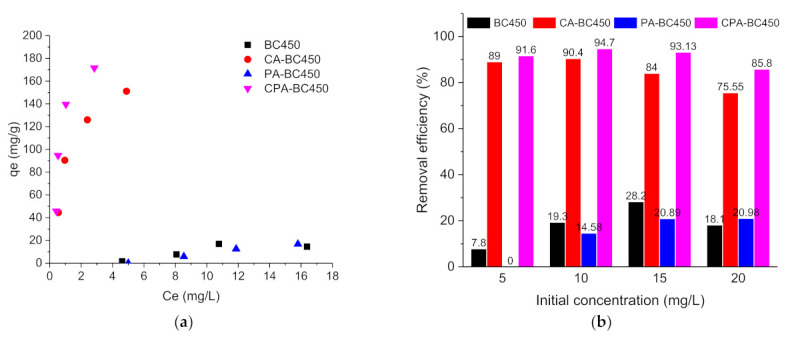

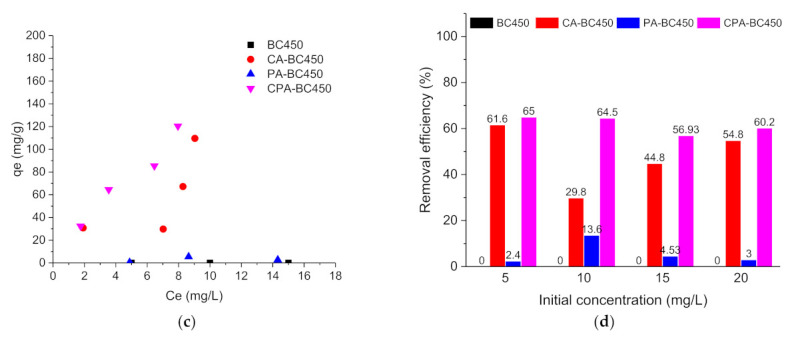
(**a**) Atrazine adsorption isotherms, (**b**) Atrazine removal efficiency (%), (**c**) Ibuprofen adsorption isotherms, and (**d**) ibuprofen removal efficiency (%) on the different adsorbents.

Atrazine or ibuprofen adsorption capacities of the different adsorbents were calculated using Equation (4), and the adsorption isotherms at room temperature are shown in Figure 11a,c, respectively. As it can be observed, the untreated char and the CO_2_-activated char exhibited very low adsorption capacities for the two adsorbates, while the KOH-activated char and KOH-CO_2_-activated char showed higher adsorption capacities for both adsorbates. The adsorption capacities of these two activated chars increased successively as the adsorbate’s concentration increased; however, saturation was not reached under the studied concentration range. The experimental data for ibuprofen adsorption on the different materials produced in this work did not follow a conventional evolution as expected. Several assumptions can be made to explain this phenomenon, such as interaction with surface of the flask, partial degradation, or low interaction with the adsorbent.

Figure 11b shows the atrazine removal efficiency as a function of the initial concentration for the different adsorbents. Upon increasing the atrazine initial concentration from 5 to 20 mg L^−1^, the atrazine removal efficiency of the CA-BC450 and CPA-BC450 samples increased slightly and reached its maximum (around 90% for CA-BC450 sample and 95% for CPA-BC450 sample) at an initial concentration of 10 mg L^−1^, and upon that value the atrazine removal efficiency decreased from 90 to 76% for CA-BC450 and from 95 to 86% for CPA-BC450. Using a BC450 or PA-BC450 sample instead, the atrazine removal was considerably lower, and it increased when the initial concentration of atrazine increased from 8 to 28% for the BC450 sample and from 0 to 21% for the PA-BC450 sample.

Figure 11d shows the ibuprofen removal efficiency as a function of the initial concentration for the different adsorbents. The removal efficiency of ibuprofen for the different adsorbents was found considerably lower than that of atrazine, and a higher removal efficiency was found for CA-BC450 and CPA-BC450 samples.

The variation trend of the adsorption properties of the different produced materials can be understood by considering their surface and chemical properties; indeed, the adsorption potential of carbon materials is essentially related to their surface area, porosity, and surface chemistry (surface functional groups), which are involved in both chemisorption and physisorption processes. Among the different tested activation methods, chemical activation produced char with a highly developed surface area and micro/meso-porosity, leading to improved adsorptive properties, whereas physical activation did not significantly enhance the textural properties of the raw char, and thus the adsorptive properties of both raw and physically activated char were comparable.

The parameters for the two adsorption isotherm models and their corresponding determination coefficients R^2^, giving the fitting of experimental data with respect to theoretical data, are presented in Table 10. The Langmuir isotherm model better describes the adsorption behavior of atrazine on CA-BC450 and CPA-BC450 samples with quite a good fit to the experimental data (R^2^ > 0.997). This indicates that the adsorption of atrazine on these adsorbents follows a monolayer model, with strong interaction between the adsorbent and the adsorbate. The maximum adsorption capacities for CA-BC450 and CPA-BC450 samples were 182 and 208 mg g^−1^, respectively, while the maximum adsorption capacities obtained using the Freundlich model were 93 and 125 mg g^−1^, respectively. K_L_ and K_F_ values for the CPA-BC450 sample were higher than for CA-BC450, indicating a higher affinity between adsorbate and adsorbent for the CPA-BC450 sample [80]. For atrazine, it is well known that the H-bond, electrostatic attraction between the hydrogen atom in the polar groups and the highly electronegative heterocyclic ring in atrazine (electron donor) and organic partitioning play an important role in the atrazine sorption processes. In general, a deeper carbonization of organic compounds with an increase in temperature may lead to more site-specific interactions during the adsorption process. However, a temperature higher than 400 °C significantly destroys the primary active sites of organic components, such as carboxylic and phenolic moieties, which are essential components for H-bonding formation for atrazine adsorption. Chemical activation might create additional adsorption sites able to create such bonds. Moreover, atrazine molecules can be easily decomposed in weak basic or acid environments, so its exposition to a higher basic environment can result in higher adsorption/degradation. Otherwise, the CA-BC450 and CPA-BC450 samples present higher surface negative charges and then can contribute to atrazine degradation.

The isotherm model parameters for ibuprofen adsorption on the CA-BC450 and CPA-BC450 samples are also shown in Table 10. The Freundlich model led to a better fit (R^2^ > 0.895) with the experimental data than the Langmuir model, which indicates that ibuprofen adsorption occurs differently on these adsorbents as compared to atrazine. The Freundlich constants (1/n) were found to be 5.124 and 0.706 for CA-BC450 and CPA-BC450, respectively, which indicates that the adsorption of ibuprofen is more favored on CPA-BC450 than on CA-BC450. From the results obtained, it is clear that the adsorption properties of the samples developed in this study towards ibuprofen were not significant when compared to atrazine. These results may be due to the surface functional groups on the activated char surface that lead to stronger interaction of the surface with atrazine than with ibuprofen.

Table 11 summarizes the results obtained for atrazine and ibuprofen adsorption on different adsorbents. No data were found for atrazine or ibuprofen adsorption on waste tires-based char or activated char. In comparison to these different studies, the activated chars developed in this study showed higher adsorption capacity for atrazine than those reported for commercial activated carbons having surface areas of 666 m^2^ g^−1^ [81] and higher than those reported for agricultural waste-based char/activated chars (hemp stem [82], soybeans [83], corn stalks [83], rice stalks [83], and corn straw [84]. On the other hand, the activated chars prepared in the present work showed weak ibuprofen adsorption properties than those reported in the literature.

## 4. Conclusions

In this work, activated carbons were successfully prepared from waste tires via pyrolysis and activation processes and employed as adsorbents of atrazine and ibuprofen as organic micro-pollutant adsorbates. Thermogravimetric analysis of ground rubber tire showed that the heating rate and pyrolysis temperature in the explored range do not seem to affect the thermal degradation of the ground rubber sample, at least from a qualitative point of view, while they sensibly influence the char yields. Pyrolysis of ground rubber samples at the different heating rates was mainly complete by 550 °C, and the maximum char yield was obtained at 450 °C with a heating rate of 5 °C min^−1^. According to the results of the char characterization, the physicochemical properties of the tire-based char samples seemed to be not too sensitive to the pyrolysis temperature. The pyrolytic chars showed high ash contents and low surface areas; therefore, activation treatments were needed to improve their properties as adsorbents. The results of the activated char characterization clearly demonstrated that the activation process led to an increase in surface area, pore volume, and surface alkalinity and a decrease in sulfur and zinc contents. The activation method has a strong influence on the evolution of the porous structure and surface structural characteristics of the resulting activated chars. The experimental results for atrazine adsorption showed that the activated chars had higher adsorption capacity than those reported in the literature. The adsorption capacity of ibuprofen on the activated chars, however, was lower than those found in the literature. Among the different tested activation methods, chemical activation produced char with a highly developed surface area and micro/meso-porosity, leading to improved adsorption properties.

## Figures and Tables

**Table 1:** Characteristics of the ground rubber feedstock.

Proximate Analysis (wt.%, as Received)	Analysis Method	Result
Moisture	Thermogravimetric Analysis (TGA)	0.4
Volatile matter		67.5
Fixed carbon		27.3
Ash		4.7
**Elemental Analysis (mg kg^−1^, dry basis)**		
As	Inductively Coupled Plasma Mass Spectrometry (ICP-MS)	0.05
Cd		0.05
Cr total		<0.25
Pb		4.2
Mn		1
Se		<0.5
Ba		7.5
B		1.5
Sb		<0.5
Co		<0.05
Sr		0.85
Sn		<0.5
Al	Inductively Coupled Plasma Atomic Emisssion Spectrometry (ICP-AES)	16.5
Cu		122
Ni		<0.25
Zn		700
Cr III	NF EN ISO 11885	<0.25
Cr VI	NF T 90-043	<0.20
Hg	NF EN ISO 17852	<0.005
**Total Polycyclic Aromatic Hydrocarbon (mg kg^−1^, dry basis)**	US EPA 8270	<43.26

**Table 2:** Details of the TGA experimental protocols.

Experimental Protocol for Non-Isothermal Tests: Study of the Influence of Heating Rate		
Step	Description	Stage
1	hold for 10 min at 35 °C under N_2_	initial
2	ramp to 900 °C with ramp rate of 5, 10 and 20 °C min^−1^ under N_2_	heating
3	hold at 900 °C for 10 min under N_2_	isothermal
4	hold at 900 °C for 60 min under air	isothermal
5	cool to room temperature under air	cooling
**Experimental Protocol for Isothermal Tests: Study of the Influence of Pyrolysis Temperature**		
**Step**	**Description**	**Stage**
1	hold for 10 min at 35 °C under N_2_	initial
2	ramp to T pyrolysis with ramp rate of 5 °C min^−1^ under N_2_	heating
3	hold at T pyrolysis for 3 h under N_2_	isothermal
4	ramp to 900 °C with ramp rate of 5 °C min^−1^ under N_2_	heating
5	hold at 900 °C for 60 min under N_2_	isothermal
6	hold at 900 °C for 10 min under air	isothermal
7	cool to room temperature under air	cooling

**Table 3:** Molecules used as micro-pollutant adsorbates for the liquid phase adsorption experiments.

Adsorbates	Type	Molecule Structure	Molecule Size *	Solubility in Water (mg L^−1^)
Atrazine C_8_H_14_ClN_5_	Herbicide	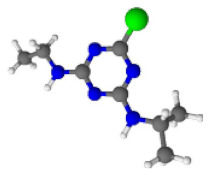	Width ≈ 1 nm Depth ≈ 0.8 nm	21 (at 25 °C)
Ibuprofen C_13_H_18_O_2_	Anti-inflammatory drug	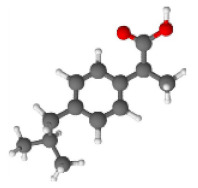	Width ≈ 1.3 nm Depth ≈ 0.5 nm	33 (at 25 °C)

* Determined by ACD/ChemSketch software.

**Table 4:** Effect of the heating rate and the pyrolysis temperature on the proximate analysis of the ground rubber sample.

Heating Rate (°C min^−1^)	Volatiles (wt.%)	Fixed Carbon (wt.%)	Ash (wt.%)	Char Yield (wt.%)
5	67.8	27.5	4.7	32.2
10	65.8	31.4	2.7	34.2
20	66.9	29.2	4.0	33.1
**Temperature (°C)**	**Volatiles (wt.%)**	**Fixed Carbon (wt.%)**	**Ash (wt.%)**	**Char Yield (wt.%)**
450	60.3	26.3	13.4	39.7
500	63.0	35.8	1.2	37.0
550	65.2	34.0	0.8	34.8
600	64.4	30.5	5.1	35.6

**Table 5:** Comparison of product yields derived from ground rubber pyrolysis reported in the literature with the results of the present study.

Reactor Type	Temperature (°C)	Solid Yield (wt.%)	Liquid Yield (wt.%)	Gas Yield (wt.%)	Reference
Pilot-scale static bed	450	38.1	29.7	32.2	This study
	500	37.8	30.5	31.7	
	550	37.8	34.2	28.0	
Laboratory-scale static bed batch	400	55.9	24.8	19.3	[12]
	500	44.8	38.0	17.2	
	600	44.2	38.2	17.5	
Pilot-scale continuous rotary kiln	450	43.9	43.0	13.1	[64]
	500	41.3	45.1	13.6	
	550	39.9	44.6	15.5	
Pilot-scale static bed batch	450	37.4	58.1	4.5	[65]
	500	38.3	56.2	5.5	
	600	38.0	53.1	8.9	
Pilot-scale fluidized bed	500	30.0	65.0	5.0	[66]
	550	34.0	57.0	9.2	
	600	40.0	51.0	9.1	

**Table 6:** Comparison of activated carbon yield reported in the literature with those obtained in the present study.

Rubber Size (mm)	Temperature (°C)/Heating Rate (°C min^−1^)	Residence Time (min)	Activating Agent/Weight Ratio	Activated Carbon Yield (wt.%)	Reference
1.5–4	700/5	180	KOH/(1:3)	70	Present study
1.5–4	850/5	120	CO_2_	87	Present study
1.5–4	700/5 and 700/5	180 and 20	KOH/(1:3) and CO_2_	57	Present study
1.1–1.7	700/3	60	KOH/(1:2)	81	[68]
1.1–1.7	750/3	60	KOH/(1:4)	74	[68]
1.5–2.0	900/N.M	120	CO_2_	93	[69]
N.M	875/20	100	CO_2_	88	[40]
0.5–1	850/N.M	120	CO_2_	62	[70]

**Table 7:** Physicochemical properties of the ground rubber-based chars.

	BC450	BC500	BC550
Proximate Analysis (wt.%, dry basis)			
Total volatile matter	9.3	8.1	4.1
Fixed carbon	78.0	79.5	86.5
Ash	12.7	12.5	9.4
Elemental analysis * (wt.%, dry basis)			
C	86.69	88.05	90.90
O	6.41	5.59	4.10
Mg	0.10	0.11	0.12
Al	0.11	0.11	0.22
Si	2.81	2.05	0.63
P	0.02	0.02	0.02
S	1.66	1.66	1.67
Cl	0.02	0.04	0.04
K	0.04	0.04	0.04
Ca	0.07	0.04	0.11
Fe	0.04	0.05	0.04
Co	0.03	0.03	0.05
Zn	1.92	2.15	1.95
Br	0.06	0.06	0.06
**Textural Properties**			
BET surface area (N_2_) (m^2^ g^−1^)	81.87	85.88	71.41
Total pore volume (N_2_) (cm^3^ g^−1^)	0.55	0.58	0.53
Average pore width ** (N_2_) (nm)	27.16	34.05	34.35
BET surface area (CO_2_) (m^2^ g^−1^)	57.08	76.15	63.58
**ID/IG ratio**	0.63	0.89	1.30

* Element concentrations ≤0.01 wt.% are not provided in the table. ** BJH desorption.

**Table 8:** Physicochemical properties of the activated chars.

Elemental Analysis (wt.%, Dry Basis)	CA-BC450	PA-BC450	CPA-BC450
C	95.96	86.90	96.13
O	1.70	6.36	1.60
Mg	0.16	0.07	0.17
Al	0.27	0.48	0.24
Si	0.64	3.11	0.59
P	0.00	0.02	0.00
S	0.26	1.23	0.22
Cl	0.02	0.00	0.00
K	0.73	0.04	0.80
Ca	0.11	0.08	0.11
Fe	0.04	0.06	0.05
Co	0.03	0.02	0.03
Cu	0.00	0.01	0.01
Zn	0.00	1.60	0.00
Br	0.01	0.01	0.00
Zr	0.02	0.01	0.01
**Textural Properties**			
BET surface area (N_2_) (m^2^ g^−1^)	493.75	164.33	490.28
Total pore volume (N_2_) (cm^3^ g^−1^)	0.48	0.72	0.50
Average pore width (N_2_) (nm)	14.97	35.12	13.35
BET surface area (CO_2_) (m^2^ g^−1^)	308.54	117.69	279.33
**ID/IG Ratio**	1.41	1.44	1.40

**Table 9:** Comparison of BET surface areas of activated chars produced in the present study with those reported in the literature.

Rubber Size (mm)	Activation Temperature (°C)/Heating Rate (°C min^−1^)	Residence Time (min)	Activating Agent/Weight Ratio	BET Surface (m^2^g^−1^)/Mesopore Volume (cm^3^g^−1^)	Reference
1.5–4	-	-	-	81/0.55	This study
1.5–4	700/5	180	KOH/(1:3)	494/0.48	This study
1.5–4	850/5	120	CO_2_	164/0.72	This study
1.5–4	700/5 and 700/5	180 and 20	KOH/(1:3) and CO_2_	490/0.50	This study
1.1–1.7	700/3	60	KOH/(1:2)	166/0.56	[68]
1.5–2	700/20	120	KOH/(1:3)	170/0.69	[71]
1.1–1.7	750/3	60	KOH/(1:4)	405/0.61	[68]
1.5–2.0	900/N.M	120	CO_2_	91/N.M	[69]
N.M	875/20	100	CO_2_	239/N.M	[40]
0.5–1	850/N.M	120	CO_2_	399/0.26	[70]

**Table 10:** Isotherm model parameters obtained from the adsorption data.

Adsorbate	Adsorbent	Isotherm Model	Parameters		R^2^
Atrazine	CA-BC450	Langmuir	q_max_ (mg g^−1^), K_L_ (L mg^−1^)	181.8, 1.000	0.999
		Freundlich	K_F_ (mg g^−1^), n	92.7, 3.148	0.991
	CPA-BC450	Langmuir	q_max_ (mg g^−1^), K_L_ (L mg^−1^)	208.3, 1.714	0.997
		Freundlich	K_F_ (mg g^−1^), n	125.0, 2.925	0.915
Ibuprofen	CA-BC450	Freundlich	K_F_ (mg g^−1^), n	0.001, 0.195	0.999
	CPA-BC450	Freundlich	K_F_ (mg g^−1^), n	25.6, 1.416	0.895

**Table 11:** Various studies for atrazine and ibuprofen adsorption in aqueous media by various adsorbents.

Adsorbate	Adsorbent	Particle Size (mm)	BET (m^2^·g^−1^)	Adsorbent Dosage	Concentration (mg L^−1^)	T	q_max_ (mg·g^−1^)	Reference
Atrazine	Waste tire-AC *	0.25−0.4	490	4 mg/40 mL	5−20	18	208	This study
Atrazine	Hemp stem-AC	0.5−1.0	2135	5–100 mg/100 mL	30	N.M	200	[81]
Atrazine	Commercial-AC	N.M	666	10 mg	2−25	N.M	30	[82]
Atrazine	Soybeans-BC **	<0.6	17.5	200 mg/100 mL	5−35	18	1.4	[83]
Atrazine	Corn stalks-BC	<0.6	19.6	200 mg/100 mL	5−35	18	1	[83]
Atrazine	Rice stalks-BC	<0.6	25.8	200 mg/100 mL	5−35	18	1.2	[83]
Atrazine	Corn straw-BC	<2	45	50 mg/100 mL	5−25	25	7	[84]
Ibuprofen	Waste tire-AC	0.25−0.4	490	4 mg/40 mL	5−20	18	-	This study
Ibuprofen	Cork waste-AC	N.M	891	10 mg/15 mL	20−60	30	85	[85]
Ibuprofen	PET-AC	N.M	1426	2.5–10 mg	20–120	30	255	[86]
Ibuprofen	Commercial-AC (Coal)	N.M	1156	2.5–10 mg	20–120	30	360	[86]
Ibuprofen	Commercial-AC (Wood)	N.M	899	2.5–10 mg	20–120	30	275	[86]

* Activated carbon. ** Biochar.

## Data Availability

All the data are contained within the article.
